# AlN/FeNi Microwave-Attenuating Ceramics with High-Efficiency Thermal Conductivity and Microwave Absorption

**DOI:** 10.3390/ma18020367

**Published:** 2025-01-15

**Authors:** Yuanwei Lin, Hetuo Chen, Longfei Wang, Liqiong An, Xianpeng Qin, Guohong Zhou

**Affiliations:** 1 State Key Laboratory of High Performance Ceramics and Superfine Microstructure, Shanghai Institute of Ceramics, Chinese Academy of Sciences, Shanghai 200050, China201930410079@stu.shmtu.edu.cn (L.W.); xpqin@mail.sic.ac.cn (X.Q.);; 2 College of Ocean Science and Engineering, Shanghai Maritime University, Shanghai 201306, China; 3 Center of Materials Science and Optoelectronics Engineering, University of Chinese Academy of Sciences, Beijing 100049, China

**Keywords:** maxwell theory, AlN/FeNi composites, microwave absorption, high thermal conductivity

## Abstract

The integration, miniaturization, and high frequency of microwave vacuum electronics put forward higher requirements for heat-conducting and wave-absorbing integrated materials. However, these materials must balance the dispersion and isolation of wave-absorbing components to optimize absorption while maintaining the continuity of thermal conductivity pathways with low defect rates and minimal interfaces. This presents a significant challenge in achieving both high thermal conductivity and efficient wave absorption simultaneously. Here, AlN/FeNi microwave-attenuating ceramics were synthesized via non–pressure sintering in a nitrogen atmosphere. The influence of FeNi content (0–20 wt%) on the density, phase composition, microstructure, microwave-absorption properties and thermal conductivity of the composites was investigated. AlN/FeNi composites consist primarily of an AlN phase with FeNi_0.0578_, Fe, AlYO_3_, and Al_5_Y_3_O_12_ as secondary phases, and the microstructure is uniform and dense. As the FeNi content rises from 0 to 20 wt%, the density of the composites sintered at 1800 °C × 2 h increases from 3.3 to 3.7 g/cm^3^. Their X-band (2–18 GHz) dielectric constant goes up from 6.5 to 8.5, the dielectric loss factor rises from 0.1 to 0.9, and thermal conductivity diminishes from 130 to 123 W/m·K. Upon reaching an FeNi content of 20 wt%, the composite achieves a minimum reflection loss of −39.1 dB at 9.5 GHz, with over 90% absorption across an effective absorption bandwidth covering 2.5 GHz. It exhibits excellent impedance matching, electromagnetic wave-attenuation properties, a relative density of 98.6%, and a thermal conductivity of 123 W m^−1^ K^−1^. The prepared AlN/FeNi composites, with integrated outstanding microwave-absorption capabilities and thermal conductivity, holds great promise for applications in 5G communications, aerospace, and artificial intelligence.

## 1. Introduction

High-speed data transmission equipment [1], key automotive components [2], portable devices like mobile intelligent terminals [3], and high-power equipment [4] necessitate microwave-attenuating ceramics for microwave vacuum electronics that possess both superior electromagnetic wave-absorption capabilities and high thermal conductivity. Microwave-attenuating ceramics leverage the medium loss mechanism to effectively diminish incident electromagnetic waves, converting their energy into heat. This heat is rapidly dissipated to the external environment via the heat conduction system.

Microwave-attenuating ceramics typically consist of thermal conductive matrix materials and microwave-absorbing agents. Currently, widely used matrix materials include alumina (Al_2_O_3_, [5]), boron nitride (BN, [6]), beryllium oxide (BeO, [7]), silicon nitride (Si_3_N_4_, [8]), and aluminum nitride (AlN, [9]). However, the low thermal conductivity of Al_2_O_3_, anisotropic thermal properties of hexagonal BN, and toxicity of BeO have rendered them unsuitable for meeting evolving development requirements. Additionally, because the structure of Si_3_N_4_ is more complex than that of AlN, leading to greater phonon scattering, the thermal conductivity of sintered Si_3_N_4_ ceramics in current studies is lower than that of AlN [10]. AlN boasts high bonding strength, minor non–harmonic lattice vibrations, and maintains a thermal conductivity comparable to BeO. With the thermal conductivity of a single AlN crystal being up to 320 W m^−1^ K^−1^ [11], AlN facilitates rapid and efficient heat dissipation from the device to its surroundings, thus preventing overheating from heat accumulation. However, AlN itself hardly absorbs microwaves, necessitating the selection of appropriate microwave absorbers.

The performance of microwave absorbers is typically enhanced by increasing material loss. According to the Maxwell theory, material loss can be expressed as follows [12]:

(1) $$tan\delta =\frac{\varepsilon^{\prime \prime }}{\varepsilon^{\prime }}+\frac{\sigma }{\omega \varepsilon^{\prime }}$$

where *ε*′ represents the real part of complex permittivity, *ε*″ is the imaginary part of complex permittivity, *σ* is the electrical conductivity, and *ω* is the angular frequency of the electromagnetic wave. Both dielectric loss and conductivity loss contribute to material loss. Table 1 outlines the key features of commonly used microwave attenuators with high thermal conductivity. These attenuators are characterized by a moderate and adjustable dielectric constant ranging from 8 to 20, which effectively prevents issues such as impedance mismatch that can arise from excessively high dielectric constants.

Dielectric loss-type wave absorbers are generally made of materials like SiC [13]. To improve the material loss, dielectric loss absorbers are required to introduce lattice defects, which can impede heat conduction, making it difficult to achieve materials with both high thermal conductivity and superior microwave-absorption characteristics. Meanwhile, the covalent nature of SiC and AlN, both belonging to the hexagonal crystal system and P63mc (186) space group, facilitates the formation of SiC–AlN solid solutions, which can increase lattice defects and significantly degrade thermal conductivity. As shown in Table 1, Li et al. [14] developed AlN/SiC composites by incorporating 30 wt% SiC through plasma-activated sintering, achieving a minimum reflection loss (*RL_min_*) of −16.5 dB at 15.5 GHz, a thermal conductivity of 24.88 W m^−1^ K^−1^, and an effective absorption bandwidth of 2.6 GHz. Fang et al. [15] engineered a spherical graphite (SG) @SiC/AlN attenuator with a core–shell structure and adjustable SiC shell thickness, fabricated via hot-pressed sintering. This resulted in an *RL_min_* of −34.2 dB at 10.99 GHz and a thermal conductivity of 63.92 W m^−1^ K^−1^. The microwave absorption and thermal conductivity of AlN/SiC composites are inadequate, necessitating the exploration of a new material system to achieve enhanced performance.

Conductive loss–type absorbers are often composed of metal particles or carbon (C) [16]. Conductive loss absorbers enhance the material loss factor by releasing free electrons. According to the Wiedemann–Franz law [17],

(2) $$\frac{k}{\sigma }=W$$

where *k* represents thermal conductivity, and *W* is a temperature–dependent constant. At a given temperature, materials with higher conductivity tend to exhibit greater thermal conductivity. Consequently, the conductive loss absorber is an optimal choice for thermal absorption materials. Fang et al. [18] enhanced thermal conductivity to 70.58 W m^−1^ K^−1^ by adding 6 wt% glassy carbon to the AlN matrix, resulting in an *RL_min_* of −22.21 dB at 11.07 GHz and a density of 98.7%. However, the properties of AlN–based thermal conductive microwave materials require further enhancement. In comparison to carbon materials, magnetic metals exhibit superior microwave-absorption characteristics. Currently, the primary magnetic metal particles include FeSiAl and FeNi [19,20]. The AlN/FeSiAl composite developed by Wang et al. [19] demonstrates an *RL_min_* of −57.31 dB at 14.32 GHz and a high thermal conductivity of 130.18 W m^−1^ K^−1^, making it suitable for microwave-attenuation applications. However, the effective absorption bandwidth (EAB) of the AlN/FeSiAl composite spans merely 1.48 GHz, necessitating further enhancement. It has been demonstrated that FeNi exhibits exceptional microwave-absorption properties and thermal conductivity. Cao et al. [20] achieved an *RL_min_* of −46.8 dB at 6.32 GHz by constructing a three-dimensional conductive network of FeNi and dual heterointerface polarization. Simultaneously, the thermal conductivity of the FeNi alloy produced by G. Ya. Khadzhay et al. [21] using electric consolidation method achieved ~100 W m^−1^ K^−1^. However, despite the high density and poor thermal stability of FeNi, it can be transformed into microwave-attenuation materials with lightweight and high thermal stability. Weng et al. [22] synthesized a FeNi/C composite that achieved an *RL_min_* of −49.90 dB at 14.48 GHz through the carbon loading of FeNi alloy particles, and the effective absorption bandwidth reached 7.23 GHz. However, research into its thermal conductivity has yet to be undertaken. Additionally, the low thermal stability of carbon-based composites results in high porosity, which restricts their use in microwave vacuum devices. In contrast, aluminum nitride, with its low density, high thermal conductivity, and superior chemical stability, is an ideal substrate material for microwave attenuators. The integration of FeNi metal particles with AlN is not only expected to enhance the composite’s loss and impedance matching, thereby improving its wave-absorption capabilities, but also establishes an efficient phonon/electron transport pathway to form a thermal conduction path, endowing the composite with excellent thermal conductivity. Consequently, a systematic investigation into the microstructure and properties is essential to develop AlN/FeNi microwave-attenuating ceramics with high-efficiency thermal conductivity and microwave absorption.

This study aims to fabricate AlN/FeNi microwave-attenuating ceramics with high-efficiency thermal conductivity and microwave absorption using pressureless sintering. The influence of FeNi content on the density, phase composition, microstructure, microwave-absorption properties, and thermal conductivity of the composites was systematically investigated.

**Table 1:** The main properties of microwave attenuators made of different types of microwave absorbers.

Absorbent Type	Main Component	Synthesis Method	*λ* (W m^−1^ K^−1^)	*ε’*	*RL_min_* (dB)	EAB (GHz)	Refs.
Dielectric loss-type	AlN/SiC	Plasma-activated sintering (1800 °C)	24.88	17	−16.5	2.6	[14]
	SG@AlN/SiC	Hot-pressed sintering (1900 °C, 25 MPa)	63.92	11.8	−34.2	/	[15]
Conductivity loss-type	AlN/GC	Hot-pressed sintering (1850 °C, 30 MPa)	70.58	20	−22.21	0.5	[18]
	AlN/FeSiAl	Pressureless sintering (1800 °C)	130.18	8	−57.31	1.48	[19]
	FeNi@MoS_2_@CoS_2_	MoS_2_ crystals with FeNi nanoparticles on the surface were grown on CoS_2_ microspheres	/	8	−46.8	6.32	[20]
	FeNi	Electroconsolidation	100	/	/	/	[21]
	FeNi/C	Coprecipitation	/	12	−49.90	7.23	[22]

## 2. Experimental Procedure

### 2.1. Sample Preparation

**Figure 1:**
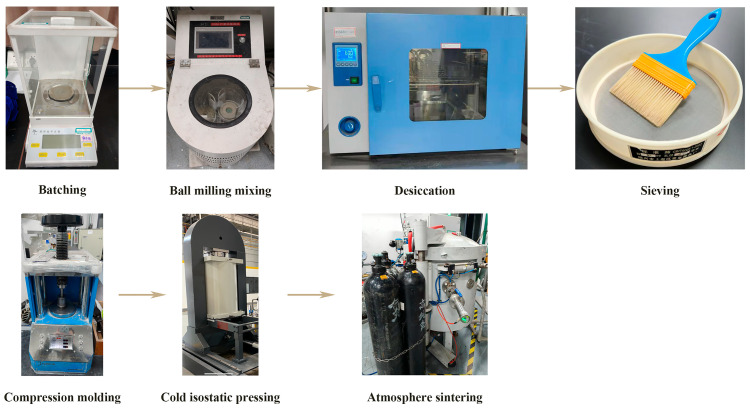
Experimental flow chart.

AlN (Tokuyama soda., Tokyo, Japan, 99.8%, Grade H), FeNi (NiWire Industries Co., Ltd., Shanghai, China, 99%, LR), and Y_2_O_3_ (Jiangyin Jiahua new material resources Co., Ltd., Jiangyin, China, 99.9%, GR) were used as raw materials. As shown in Figure 1, FeNi was added to the AlN matrix at 0, 5, 10, 15, and 20 wt%, respectively, with 4 wt% Y_2_O_3_ serving as a sintering aid. The powders were mixed thoroughly using ethanol (the mass ratio of powder to ethanol was 1:1) as a grinding medium in a roller mixer at 220 r/min for 24 h. After drying, the mixed powder was screened through an 80-mesh sieve. Green compacts were formed under a pressure of 20 MPa, and their density was further enhanced by cold isostatic pressing at 200 MPa. The AlN/FeNi composites were prepared in a 99.999% high-purity nitrogen atmosphere with a controlled heating rate of 10 °C/min and a temperature of 1800 °C for 2 h.

### 2.2. Sample Characterization

The bulk density (*ρ_v_*) of the AlN/FeNi composites was characterized using the Archimedes method (JA3003, Sainyu Hengping Instrument, Shanghai, China). The formula for calculating the bulk density of the sample was as follows: $\rho =m_{1}/(m_{2}-m_{3})\rho_{1}$. Among them, *m*_1_ was the mass of the sample after drying. After the sample had been boiled in deionized water for 3 h, *m*_2_ denoted the saturated mass of the sample once it had cooled and been wiped dry. The sample was suspended in deionized water after cooling and boiling, and the floating weight of the sample was measured to be *m*_3_. *ρ*_1_ was the density of the deionized water in the tank, determined using the drainage method during the experiment, with the density of deionized water at room temperature being approximately 1 g cm^−3^. The relative density (*ρ_r_*) was calculated by comparing the measured volume density to the theoretical density (*ρ_t_*), following the mixing rule $\rho_{r}=\rho_{v}/\rho_{t}$. The phase composition of the composites was determined via X–ray diffraction (XRD, D/Max2550V, NEO Confucianism, Tokyo, Japan) with Cu–Kα radiation, scanning from 2*θ* = 10–80° at a rate of 2 min^−1^. The microstructure was observed using a scanning electron microscope (SEM, Hitachi TM3000, Tokyo, Japan) equipped with energy dispersive spectroscopy (EDS). The thermal diffusion coefficient (*D*) was measured with a laser scatterometer (LFA467 Nanoflash, Netzsch Instruments Co. Ltd., Selb, Germany), and the specific heat capacity (*C_p_*) was determined using a differential scanning calorimeter (DSC8000, PerkinElmer, Waltham, MA, USA). The thermal conductivity (*λ*) was calculated using the following formula [23]:

(3) $$\lambda =D\rho C_{p}$$

A vector network analyzer (VNA, N5227B, Agilent, Santa Clara, CA, USA) was employed to measure the complex permittivity of the sample within the frequency range of 0.5–18 GHz. Electromagnetic measurements were conducted on standard-sized ring samples with an outer diameter of 7.00 mm, an inner diameter of 3.04 mm, and a length of 2.00 mm.

## 3. Results and Discussion

### 3.1. Density, Phase Composition, and Microstructure

**Figure 2:**
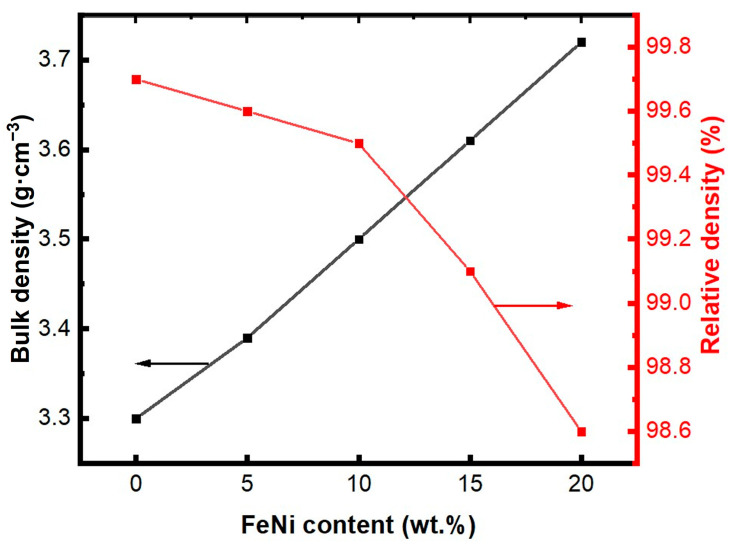
Bulk density and relative density of AlN/*x* wt% FeNi composites.

Figure 2 illustrates the density and relative density curves for AlN/*x* wt% FeNi composites. As depicted, the bulk density of the composites increases with the addition of FeNi alloy. This increase is attributed to FeNi’s higher density compared to AlN. Conversely, the inclusion of FeNi alloy also results in a reduction in the AlN/FeNi composites’ relative density. This is likely due to the inherent heterogeneity between FeNi and AlN, which creates non-uniform interfaces and diminishes the degree of densification, thereby increasing material loss. Nevertheless, due to the high diffusivity of the FeNi alloy, it enhances the mass transfer of the composite materials during the sintering process, facilitating densification. Additionally, the formation of Y-Al-O low-melting point compounds between FeNi and AlN grains promotes the bonding between these grains. Consequently, when the FeNi alloy content reaches 20%, the AlN/FeNi composite retains a high relative density of 98.6%.

**Figure 3:**
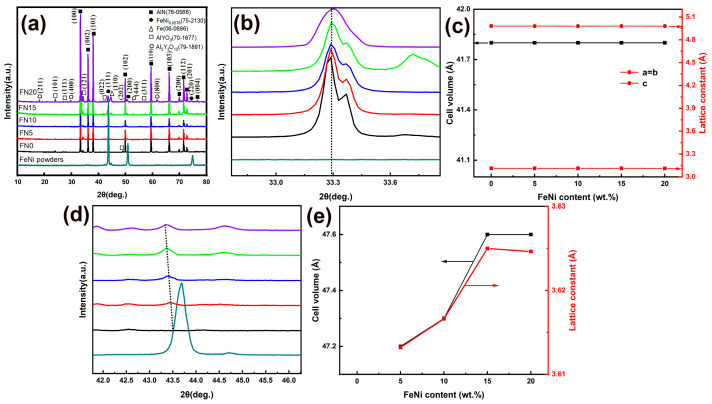
XRD patterns of FeNi powders and AlN/*x* wt% FeNi composites: (**a**) XRD patterns; (**b**–**e**) the local amplification of the XRD pattern and the corresponding cell volume and lattice constant curves, respectively.

Figure 3 depicts the XRD patterns of AlN/*x* wt% FeNi composites. As depicted, the ceramic predominantly consists of AlN (PDF card: 76–0566), with FeNi_0.0578_ (PDF card: 75–2130), Fe (PDF card: 06–0696), AlYO_3_ (PDF card: 70–1677), and Al_5_Y_3_O_12_ (PDF card: 79–1891) as secondary phases (Figure 3a). Figure 3b and Figure 3d, respectively, illustrate the variation in the primary crystal plane (100) of the AlN phase and the FeNi_0.0578_ primary crystal plane (111) with increasing FeNi addition. Figure 3c,e depicts the corresponding changes in the cell volume and lattice constant of the AlN phase and FeNi_0.0578_, as determined by XRD Rietveld refinement. As the FeNi content increases, the XRD peaks and cell parameters of AlN remain virtually unchanged. This indicates that FeNi and Y_2_O_3_ exhibit minimal reactivity with AlN, which is beneficial for preserving the continuity of the AlN lattice and maintaining the material’s high thermal conductivity. In conjunction with the Bragg diffraction equation (2dsinθ=nλ), where *d* represents the crystal plane spacing, *θ* is the angle between the incident ray and the reflecting crystal plane, *λ* is the wavelength, and *n* is the order of reflection, it is observed that with increased FeNi doping, the XRD peaks of FeNi shift towards smaller angles, the crystal plane spacing increases, and, consequently, the cell volume and cell constants expand. This phenomenon can be attributed to the high diffusivity of FeNi, which facilitates the dissolution of ions from AlN and Y_2_O_3_ into the FeNi lattice, leading to lattice expansion. During the sintering process, Y_2_O_3_ can react with the Al_2_O_3_ on the surface of AlN powder to form low-melting-point AlYO_3_ and Al_5_Y_3_O_12_. The reaction equation is as follows:

(4) $$2Y_{2}O_{3}+{3Al}_{2}O_{3}\to AlYO_{3}+{Al}_{5}Y_{3}O_{12}$$

Consequently, Y_2_O_3_ acts not only as a sintering aid to promote mass transfer and achieve densification but also serves to reduce oxygen defects within the AlN grains, making it the preferred sintering aid for AlN matrix composites.

**Figure 4:**
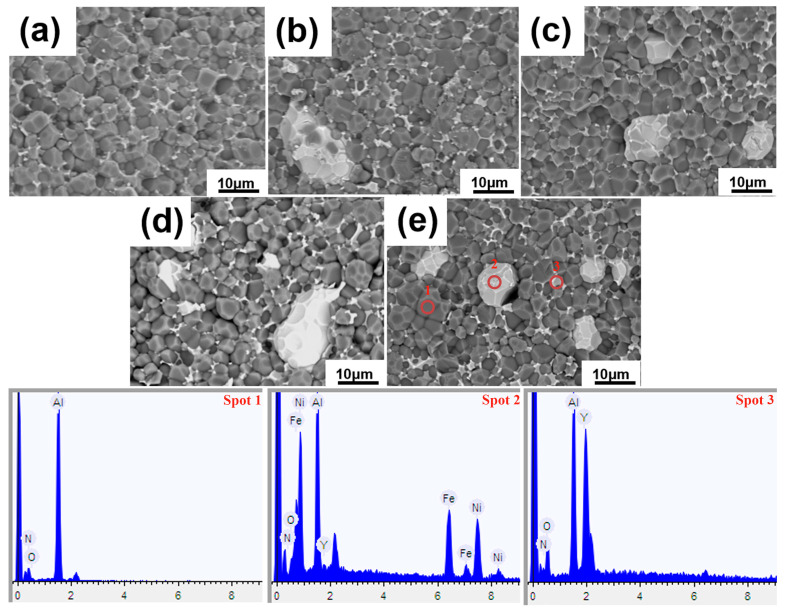
SEM images and EDS spectra of AlN/*x* wt% FeNi composites, *x* = (**a**) 0, (**b**) 5, (**c**) 10, (**d**) 15 and (**e**) 20.

Figure 4 presents SEM images and EDS spectra of AlN/*x* wt% FeNi composites. The irregular, massive FeNi alloy particles are uniformly distributed within the AlN matrix with fewer pores, which is consistent with the above-mentioned density results and further confirms that the sintering system is reasonable. When combined with EDS spectra, it becomes evident that a small quantity of oxygen ions from Y_2_O_3_ and nitrogen ions from AlN have undergone mutual diffusion. Given the high lattice activity of FeNi at elevated temperatures, a portion of AlN and Y_2_O_3_ is solubly diffused into the FeNi lattice. This process, while detrimental to the material’s thermal conductivity, is beneficial for enhancing dielectric loss and improving microwave performance. Under the action of sintering additives, the formation of a Y-Al-O liquid phase between AlN and FeNi grains promotes grain bonding, which is conducive to the development of a dense microstructure.

### 3.2. Microwave-Absorption Properties

**Figure 5:**
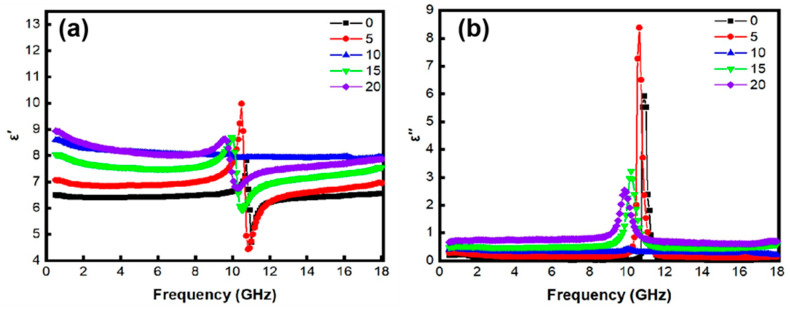
Complex permittivity curves of AlN/*x* wt% FeNi composites: (**a**) real part *ε*′, (**b**) imaginary part *ε*″.

Figure 5 illustrates the complex permittivity ($\varepsilon_{r}=\varepsilon^{\prime }-j\varepsilon^{\prime \prime }$) of AlN/*x* wt% FeNi composites. The real part (*ε*′) of the complex dielectric constant typically represents energy storage, whereas the imaginary part (*ε*″) primarily signifies energy dissipation [24]. In the frequency range of 2–18 GHz, with the increase in FeNi content, the complex permittivity of AlN/FeNi composites increases in both real and imaginary parts. Undoped AlN ceramics exhibit a low dielectric constant and low loss, aligning with previous findings [19]. As the FeNi content rises to 20 wt%, the *ε*’ value of the AlN/FeNi composite increases from 6 to 9, and the *ε*’’ value increases from 0.1 to 0.9 across the X–band, exhibiting frequency independence. The dielectric constant of ceramics doped with 20 wt% FeNi does not exceed 10, with the imaginary part of the dielectric constant being approximately 0.9, corresponding to a dielectric loss of about 0.1, which significantly enhances microwave-absorption performance. According to Lichtenecker formula [25], the composite’s dielectric constant can be correlated with the dielectric constants of its individual phases and their respective volume fractions. The elevated permittivity of FeNi, along with its increased relative content, contributes to the enhancement of the permittivity in AlN/FeNi composites. On the one hand, a portion of the AlN and Y_2_O_3_ dissolves into the FeNi lattice, forming a solid solution that induces the creation of dipoles and defects, resulting in dipole polarization loss. On the other hand, in the metal/ceramic system, charge accumulation at the metal/ceramic interface occurs when an electric field is applied, resulting in interface polarization, thus enhancing the polarization loss, which enhances the microwave-absorption performance. FeNi alloys provide rapid pathways for electron transport, leading to conductivity losses. The polarization relaxation loss in Equation (1) is frequency sensitive, whereas conductivity loss is frequency independent in the X-band. With increasing FeNi content, isolated FeNi particles tend to aggregate and form conductive clusters. The *ε*″ value of AlN containing 20 wt% FeNi exhibits a high value across the X-band, suggesting that conductivity loss is the primary loss mechanism for isolated FeNi metal particles within the AlN matrix.

**Figure 6:**
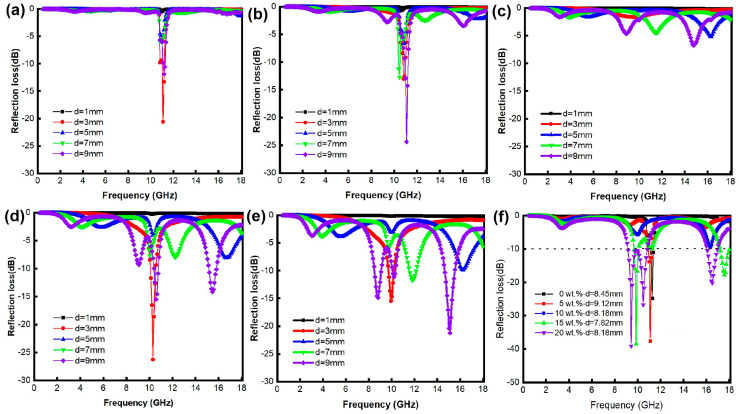
Reflection loss curve of AlN/*x* wt% FeNi composites, (**a**) *x* = 0, (**b**) *x* = 5, (**c**) *x* = 10, (**d**) *x* = 15, (**e**) *x* = 20. (**f**) Reflection loss curve of AlN/*x* wt% FeNi composites at the optimal thickness.

Figure 6 displays reflection loss curves for the AlN/*x* wt% FeNi composites. It is evident that the microwave-absorption properties of the AlN/FeNi composites are significantly influenced by the thickness, frequency, and FeNi content. Testing the reflection loss of AlN/FeNi composites at various thicknesses reveals that the reflection loss is more pronounced when the thickness is approximately 7–9 mm (Figure 6a–e). By fine-tuning the sample thickness, reflection loss curves for the optimal thickness at different FeNi contents were obtained, as depicted in Figure 6f. The findings indicate that FeNi substantially enhances the reflection loss of the AlN–based composites. Notably, the composite with 20 wt% FeNi exhibits the most superior microwave-absorption capacity, achieving a minimum reflection loss of −39.1 dB with multiple absorption peaks. When reflection losses fall below −10 dB, electromagnetic absorption exceeds 90%, thus defining the effective absorption bandwidth corresponding to the frequency range below this threshold. The composite with 20 wt% FeNi boasts an effective absorption bandwidth of 2.5 GHz.

**Figure 7:**
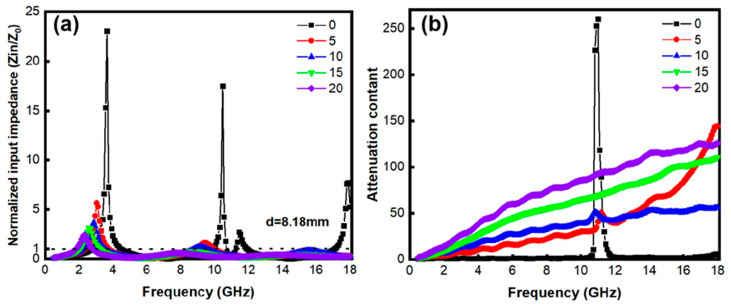
(**a**) Normalized input impedance *Z_in_*/*Z*_0_ and (**b**) attenuation coefficient of AlN/*x* wt% FeNi composites.

Figure 7 shows the normalized input impedance *Z_in_*/*Z*_0_ and absorption coefficient for the AlN/*x* wt% FeNi composites. The impedance (*Z*) and absorption coefficient (*α*) can be calculated using the following formulas [26]:

(5) $$Z_{in}=Z_{0}\sqrt{\frac{u_{r}}{\varepsilon_{r}}}tanh\left(j\frac{2\pi fd}{c}\sqrt{u_{r}\varepsilon_{r}}\right)$$

(6) $$\alpha =\frac{\pi f}{c}\sqrt{2(\mu^{\prime \prime }\varepsilon^{\prime \prime }-\mu^{\prime }\varepsilon^{\prime }+\sqrt{\left({\mu^{\prime \prime }}^{2}+{\mu^{\prime }}^{2})+({\varepsilon^{\prime \prime }}^{2}+{\varepsilon^{\prime }}^{2}\right)})}$$

where *Z_in_* and *Z*_0_ represent the impedance at the material’s surface and in free space, respectively, with *Z*_0_ being the characteristic impedance of air, which is fixed at 1. The variables *ε*, *μ*, and *d* denote the dielectric constant, permeability, and thickness of the absorbing material, while *f* and *c* are the incident electromagnetic frequency and the speed of light (3 × 10^8^ m/s), respectively. As the doping of FeNi increases, the *Z_in_*/*Z*_0_ value of the AlN/FeNi composites approaches 1, indicating improved impedance-matching performance and easier penetration of electromagnetic waves into the material. Additionally, with increased FeNi doping, the absorption coefficient rises, signifying enhanced electromagnetic wave dissipation capability. This is because each interface where AlN and FeNi come into contact can be considered a capacitive model. When an electromagnetic wave penetrates, the charge carriers migrate within this capacitor-like structure, resulting in the attenuation of the electromagnetic wave. Consequently, the composite doped with 20 wt% FeNi exhibited the optimal impedance-matching performance and electromagnetic wave attenuation.

### 3.3. Thermal Conductivity

**Figure 8:**
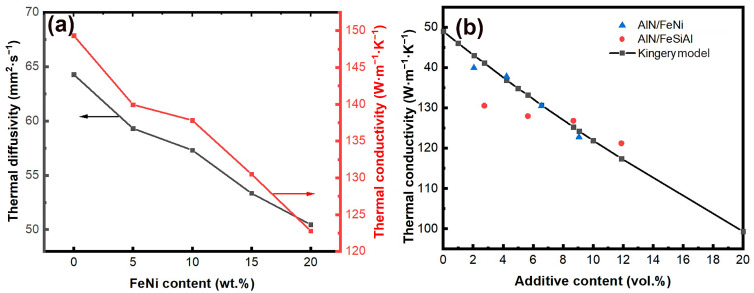
(**a**) Thermal diffusion coefficient and thermal conductivity of AlN/*x* wt% FeNi composite ceramics. (**b**) Relationship between the thermal conductivity of AlN-based composite ceramics and the volume content of absorbing agent. The AlN/FeSiAl data come from the study of Wang et al. [19].

Figure 8 illustrates the thermal conductivity properties of the AlN/*x* wt% FeNi composites. Figure 7a depicts their thermal characteristics. As the FeNi content increases, the thermal conductivity of AlN/FeNi composites declines, a result of FeNi’s lower thermal conductivity compared to AlN. Nonetheless, over a broad range of FeNi doping concentrations from 0 to 20 wt%, the composites retain a high thermal conductivity, consistently exceeding 120 W m^−1^ K^−1^, demonstrating outstanding thermal conductivity properties. Figure 7b displays the correlation between the thermal conductivity of AlN-based composites and the volume content of the absorbing agent FeNi. For composites, theoretical thermal conductivity can be determined using the Kingery model [27]:

(7) $$k_{i}=\sum v_{i}k_{i}$$

where *k_i_* represents the thermal conductivity of each component and $v_{i}$ is the volume fraction of each component. Upon comparison with values calculated using the Kingery model, it is observed that the discrepancy between measured and calculated values throughout the entire experimental range does not exceed 8%, indicating that the experimental thermal conductivity closely aligns with the theoretical predictions. This is consistent with results previously reported by Wang et al. [19]. The volume content of the microwave absorbent significantly influences the thermal conductivity of AlN-based composites. The theory also supports the model of a continuous distribution of the AlN matrix and an island distribution of FeNi microwave absorbent.

In summary, the enhanced loss, impedance matching, and excellent attenuation capacity of AlN/FeNi contribute to its superior absorption value and broader absorption bandwidth. The compact structure of AlN/FeNi and the continuity of the AlN matrix ensure the high thermal conductivity of the composite. These results are compared with relevant data reported in the previous literature, as shown in Table 2.

## 4. Conclusions

To meet the demands of integration, miniaturization, and high-frequency operation in microwave vacuum electronic devices, there is an increasing need for materials that can efficiently conduct heat and absorb microwaves. In this study, we fabricated AlN-based thermal-conductive microwave-absorbing composites using iron-based alloy (0–20 wt% FeNi) as the microwave absorber. The density, phase composition, microstructure, microwave-absorption properties, and thermal conductivity of AlN/FeNi composites were systematically investigated, leading to the following conclusions:

(1) By tuning the FeNi content, we achieved strong absorption across various frequency bands.
(2) The incorporation of FeNi did not notably compromise the thermal conductivity of the AlN matrix, resulting in the production of AlN/FeNi composites with high thermal conductivity.
(3) The composite doped with 20 wt% FeNi demonstrated the optimal microwave-absorption performance, impedance-matching characteristics, electromagnetic wave-attenuation capacity, and high thermal conductivity.

The excellent properties of AlN/FeNi composites position them as a promising candidate for thermal microwave absorption materials.

## 5. Outlook

The thermal conductivity and wave-absorption properties of AlN/FeNi composites can be enhanced by tailoring their morphology, structure, and distribution. Despite significant progress, there remains deficiencies in both research and application. The development of AlN-based integrated materials can be pursued in three key areas:

(1) Multi-functional materials: Develop thermally conductive AlN-based integrated materials with hydrophobic, vibration-resistant, radiation-resistant, high-temperature-stable, and durable properties to withstand severe environments. Concurrently, focus on the advancement of self-adaptive and self-healing capabilities of the material to extend its lifespan;
(2) Sustainable development: Investigate the sustainability and eco-friendliness of thermal absorbent AlN-based materials, minimizing environmental impact through green chemical synthesis, biosynthesis, and the use of renewable materials;
(3) Expansion of applications: Thermally conductive and absorbing AlN-based materials have seen advancements in numerous fields and are poised to extend into even more fields in the future. Investigating the the interaction mechanisms and coupling effects across different physical domains and leveraging artificial intelligence can achieve efficient material design and performance prediction.

## Figures and Tables

**Table 2:** Main properties of AlN-based microwave-attenuating ceramics.

Absorbent Type	Main Component	Synthesis Method	*λ* (W m^−1^ K^−1^)	*ε*’	*RL_min_* (dB)	EAB (GHz)	Refs.
Conductivity loss-type	AlN/Mo	Hot-pressed sintering (0.12 MPa, 1850 °C)	76	24	−32	/	[28]
	AlN/W	Pressureless sintering (1850 °C)	68	15	−23	/	[29]
	AlN/ZrB_2_	Hot-pressed sintering (20 MPa, 1850 °C)	82	10	/	/	[4]
	AlN/TiB_2_	Pressureless sintering (1850 °C)	77	10.5	−5	/	[29]
	AlN/C	Hot-pressed sintering (1800 °C)	50.1	13.9	−49.4	2	[30]
	AlN/FeSiAl	Pressureless sintering (1800 °C)	130.2	8.3	−57.3	1.5	[19]
Dielectric loss-type	AlN/SiC	Hot-pressed sintering (1900 °C, 25 MPa)	/	60	−24.2	/	[31]

## Data Availability

The original contributions presented in the study are included in the article, further inquiries can be directed to the corresponding authors.

## Nomenclature

**Abbreviations**	
DSC	Differential scanning calorimeter
EDS	Energy dispersive spectroscopy
SG	Spherical graphite
SEM	Scanning electron microscope
XRD	X–ray diffraction
**Symbols**	
*ω*	Angular frequency of the electromagnetic wave
*α*	Absorption coefficient
*ρ_v_*	Bulk density
*ε_r_*	Complex permittivity
*u_r_*	Complex permeability
*Z_0_*	Characteristic impedance of air
*ρ_1_*	Density of deionized water
*m_1_*	Dry weight
*σ*	Electrical conductivity
*m_3_*	Floating weight
*εʹʹ*	Imaginary part of complex permittivity
*u’’*	Imaginary part of complex permeability
*f*	Incident electromagnetic frequency
*Z*	Impedance
*Z_in_*	Impedance at the material’s surface
*RL_min_*	Minimum reflection loss
*Z_in_/Z_0_*	Normalized input impedance
*εʹ*	Real part of complex permittivity
*u’*	Real part of the complex permeability
*ρ_r_*	Relative density
*c*	Speed of light
*m_2_*	Saturated mass
*C_p_*	Specific heat capacity
*d*	Thickness
*k*	Thermal conductivity
*k_i_*	Thermal conductivity of each component
*W*	Temperature-dependent constant
*ρ_t_*	Theoretical density
*D*	Thermal diffusion coefficient
*λ*	Thermal conductivity
*ʋ* *_i_*	Volume fraction of each component

## References

[1] Lv H., Cui J., Li B., Yuan M., Liu J., Che R. (2024). Insights into civilian electromagnetic absorption materials: Challenges and innovative solutions. Adv. Funct. Mater..

[2] Katiyar M., Prasad M., Agarwal K., Singh R.K., Kumar A., Prasad N.E. (2018). Development of low density, heat resistant and broadband microwave absorbing materials (MAMs) for stealth applications. Silicon.

[3] Huo Y., Zhao K., Miao P., Kong J., Xu Z., Wang K., Li F., Tang Y. (2020). Microwave absorption performance of SiC/ZrC/SiZrOC hybrid nanofibers with enhanced high–temperature oxidation resistance. ACS Sustain. Chem. Eng..

[4] Patel M., Reddy J.J., Prasad V.B. (2021). High thermal conductivity aluminium nitride–zirconium diboride (AlN–ZrB_2_) composite as microwave absorbing material. Ceram. Int..

[5] Fu K., Yao Q., Xu L., Zhou W., Wang Z., Yang Y., Tong G., Wang X., Wu W. (2024). Constructing magnetic/dielectric dual loss and phonon/electron thermal carriers γ–Al_2_O_3_–based yolk–shell microspheres to collaboratively advance microwave absorption and heat conduction. Mater. Horiz..

[6] Zhong X., He M., Zhang C., Guo Y., Hu J., Gu J. (2024). Heterostructured BN@ Co-C@ C endowing polyester composites excellent thermal conductivity and microwave absorption at C band. Adv. Funct. Mater..

[7] He Y., Li X., Zhang J., Li X., Duan Y., Huang M., Bai H., Jiang D., Qiu T. (2018). Method for fabricating microwave absorption ceramics with high thermal conductivity. J. Eur. Ceram. Soc..

[8] Chen H., Wang W., Yu X., Zuo K., Xia Y., Yin J., Liang H., Yao D., Zeng Y. (2020). The effect of annealing temperature on flexural strength, dielectric loss and thermal conductivity of Si_3_N_4_ ceramics. J. Alloys Compd..

[9] Fang X., Jiang L., Pan L., Yin S., Qiu T., Yang J. (2021). High–thermally conductive AlN–based microwave attenuating composite ceramics with spherical graphite as attenuating agent. J. Adv. Ceram..

[10] Watari K., Hirao K., Toriyama M., Ishizaki K. (1999). Effect of grain size on the thermal conductivity of Si_3_N_4_. J. Am. Ceram. Soc..

[11] Slack G.A. (1973). Nonmetallic crystals with high thermal conductivity. J. Phys. Chem. Solids.

[12] Krupka J. (2006). Frequency domain complex permittivity measurements at microwave frequencies. Meas. Sci. Technol..

[13] Kuang J., Xiao T., Zheng Q., Xiong S., Wang Q., Jiang P., Liu W., Cao W. (2020). Dielectric permittivity and microwave absorption properties of transition metal Ni and Mn doped SiC nanowires. Ceram. Int..

[14] Li P., Wang C., Liu H., Shen Q., Zhang L. (2019). Structural, thermal and dielectric properties of AlN–SiC composites fabricated by plasma activated sintering. Adv. Appl. Ceram..

[15] Fang X., Hou S., Pan L., Yin S., Wang Y., Li Q., Chen D., Jin J., Yang J. (2023). Core–shell spherical graphite@ SiC attenuating agent for AlN–based microwave attenuating ceramics with high–efficiency thermal conduction and microwave absorption abilities. Ceram. Int..

[16] Zhang Y., Agrawal D.K., Cheng J., Slawecki T. (2018). Microwave power absorption mechanism of metallic powders. IEEE Trans. Microw. Theory Tech..

[17] Franz R., Wiedemann G. (1853). Ueber die wärme-leitungsfähigkeit der metalle. Ann. Phys..

[18] Fang X., Pan L., Yin S., Chen H., Qiu T., Yang J. (2020). Spherical glassy carbon/AlN microwave attenuating composite ceramics with high thermal conductivity and strong attenuation. Ceram. Int..

[19] Wang L., An L., Zhou G., Wang X., Sun K., Chen H., Hou H. (2022). Dense AlN/FeSiAl composite ceramics with high thermal conductivity and strong microwave absorption. J. Mater. Sci. Mater. Electron..

[20] Cao X., Jia Z., Hu D., Wu G. (2022). Synergistic construction of three–dimensional conductive network and double heterointerface polarization via magnetic FeNi for broadband microwave absorption. Adv. Compos. Hybrid Mater..

[21] Khadzhay G.Y., Vovk S.R., Vovk R.V., Gevorkyan E.S., Zubenko N.S., Kislitsa M.V., Chishkala B.O., Feher A., Kollar P., Fuzer J. (2020). Electrical and thermal conductivity of FeNi at low temperatures. Low Temp. Phys..

[22] Weng L., Lei X., Zhang Z., Wang J. (2023). Porous carbon–loaded hollow FeNi alloy particles for highly efficient electromagnetic wave absorbing materials. Synth. Met..

[23] Zhao L., Lo S.H., Zhang Y., Sun H., Tan G., Uher C., Wolverton C., Dravid V.P., Kanatzidis M.G. (2014). Ultralow thermal conductivity and high thermoelectric figure of merit in SnSe crystals. Nature.

[24] Zhang B., Wu C., Ye K., Sun C., Wang Z. (2023). Dual–induced SiC/SME multifunction composite for high–efficiency broadband electromagnetic wave absorption. Carbon.

[25] Lichtenecker K. (1926). Die ableitung der logarithmischen mischungsregel aus dem maxwell–rayleighschen schrankenwertverfahren. Kolloidchem. Beih..

[26] Wu H., Qin M., Zhang L. (2020). NiCo_2_O_4_ constructed by different dimensions of building blocks with superior electromagnetic wave absorption performance. Compos. Part B.

[27] Kingery W.D. (1959). Thermal conductivity: XIV, conductivity of multicomponent systems. J. Am. Ceram. Soc..

[28] Chasnyk V.I., Chasnyk D.V., Fesenko I.P., Kaidash O.M.A. (2020). Study of the thermal conductivity, electrical resistivity, and microwave absorption of pressureless sintered AlN–Y_2_O_3_–Mo and AlN–Y_2_O_3_–TiN composites. J. Superhard Mater..

[29] Chasnyk V., Chasnyk D., Fesenko I., Kaidash O., Turkevych V. (2021). Dielectric characteristics of pressureless sintered AlN–based composites in the 3–37 GHz frequency range. J. Mater. Sci. Mater. Electron..

[30] Zhao J., Chen H., Yang J., Li J., Wang Z., Zhou G., Ma Z., Gu Q. (2024). Microwave absorption, thermal and mechanical properties of amorphous nano carbon doped aluminium nitride composite ceramics. Ceram. Int..

[31] Fang X., Jiang S., Pan L., Yin S., Qiu T., Yang J., Li X. (2021). β-SiC/AlN microwave attenuating composite ceramics with excellent and tunable microwave absorption properties. J. Eur. Ceram. Soc..

